# Uric Acid and Preeclampsia: Pathophysiological Interactions and the Emerging Role of Inflammasome Activation

**DOI:** 10.3390/antiox14080928

**Published:** 2025-07-29

**Authors:** Celia Arias-Sánchez, Antonio Pérez-Olmos, Virginia Reverte, Isabel Hernández, Santiago Cuevas, María Teresa Llinás

**Affiliations:** 1 Molecular Inflammation Group, University Clinical Hospital Virgen de la Arrixaca, Biomedical Research Institute of Murcia (IMIB), 30120 Murcia, Spain; celia.ariass@um.es (C.A.-S.); antonio.perezo@um.es (A.P.-O.); santiago.cuevas@um.es (S.C.); 2 Department of Physiology, Faculty of Medicine, University of Murcia, 30120 Murcia, Spain; vrr2@um.es (V.R.); isabelhg@um.es (I.H.); 3 Medical Physiology Group, University Clinical Hospital Virgen de la Arrixaca, Biomedical Research Institute of Murcia (IMIB), 30120 Murcia, Spain

**Keywords:** uric acid, preeclampsia, inflammation, NLRP3 inflammasome, oxidative stress, pregnancy, IL-1β

## Abstract

Preeclampsia (PE) is a multifactorial hypertensive disorder unique to pregnancy and a leading cause of maternal and fetal morbidity and mortality worldwide. Its pathogenesis involves placental dysfunction and an exaggerated maternal inflammatory response. Uric acid (UA), traditionally regarded as a marker of renal impairment, is increasingly recognized as an active contributor to the development of PE. Elevated UA levels are associated with oxidative stress, endothelial dysfunction, immune activation, and reduced renal clearance. Clinically, UA is measured in the second and third trimesters to assess disease severity and guide obstetric management, with higher levels correlating with early-onset PE and adverse perinatal outcomes. Its predictive accuracy improves when combined with other clinical and biochemical markers, particularly in low-resource settings. Mechanistically, UA and its monosodium urate crystals can activate the NLRP3 inflammasome, a cytosolic multiprotein complex of the innate immune system. This activation promotes the release of IL-1β and IL-18, exacerbating placental, vascular, and renal inflammation. NLRP3 inflammasome activation has been documented in placental tissues, immune cells, and kidneys of women with PE and is associated with hypertension, proteinuria, and endothelial injury. Experimental studies indicate that targeting UA metabolism or inhibiting NLRP3 activation, using agents such as allopurinol, metformin, or MCC950, can mitigate the clinical and histopathological features of PE. These findings support the dual role of UA as both a biomarker and a potential therapeutic target in the management of the disease.

## 1. Introduction

Preeclampsia (PE) is a multisystem disorder unique to pregnancy, affecting 2–8% of pregnant women worldwide. It is a major cause of maternal and fetal morbidity and mortality, contributing to over 500,000 fetal and neonatal deaths and more than 70,000 maternal deaths annually [1]. Until the last decade, PE was defined by the new onset of hypertension after 20 weeks of gestation accompanied by proteinuria ≥300 mg/day. However, in 2013 and 2014, the American College of Obstetricians and Gynecologists [2] and the International Society for the Study of Hypertension in Pregnancy [3], respectively, revised the criteria, removing the requirement for proteinuria. This change recognized that maternal and fetal risks also exist in nonproteinuric forms of PE.

Currently, PE is diagnosed by the de novo development of hypertension after 20 weeks of gestation, along with at least one of the following: proteinuria, evidence of maternal acute kidney injury, liver dysfunction, neurological symptoms, hemolysis, thrombocytopenia, or uteroplacental dysfunction [3].

Although the terms early-onset and late-onset are used to describe subtypes of PE, the precise onset of the disease is often difficult to establish. Therefore, in clinical settings, the time of delivery is used to differentiate the two forms. Early-onset PE (<34 weeks of gestation), typically more severe, is marked by low cardiac output, high vascular resistance, frequent fetal growth restriction, and preterm birth. Conversely, late-onset PE (≥34 weeks of gestation), accounting for 80–95% of global cases, is associated with high cardiac output and normal or low vascular resistance. While generally linked to lower morbidity, late-onset PE still poses long-term cardiovascular risks for both mother and child [4].

Even though its exact etiology remains debated, the most widely accepted theory argues that PE develops in two stages. The updated two-stage model proposes that all cases of PE, both early- and late-onset, share a common factor: stress of the placental syncytiotrophoblast [5]. In this model, the first stage involves placental dysfunction, due either to poor trophoblast invasion and incomplete remodeling of the spiral arteries (typical of early-onset PE) or to intrinsic compression of the intervillous space in placentas that exceed uterine capacity (common in late-onset PE) [5,6]. In both scenarios, syncytiotrophoblast stress leads to the release of abnormal placental factors into the maternal circulation, triggering a systemic maternal inflammatory response that affects vascular endothelium and initiates the clinical phase of the disease characterized by hypertension and multi-organ involvement [6].

This process is further modulated by pre-existing maternal conditions such as obesity, chronic hypertension, diabetes, autoimmune disorders, and specific immunologic characteristics of the uterine environment, all of which increase vascular sensitivity to placental pro-inflammatory mediators [6]. In PE, the maternal immune system shifts to a state of chronic inflammation with abnormal activation of both innate and adaptive immunity [7]. Immune cells, including T and B lymphocytes, NK cells, macrophages, and dendritic cells, contribute to a sustained inflammatory cascade. These cells mediate vascular injury, endothelial dysfunction, and tissue damage via the production of pro-inflammatory cytokines [8]. This exaggerated immune activation forms a positive feedback loop that maintains and amplifies inflammation, persisting even beyond pregnancy and increasing the risk of postpartum cardiovascular disease [9].

NLRP3-dependent inflammasome activation has been implicated in the inflammatory processes associated with the pathogenesis of PE [10]. This multiprotein complex is assembled in response to danger-associated molecular patterns (DAMPs) such as ATP, uric acid (UA), reactive oxygen species (ROS), cholesterol, HMGB1, extracellular vesicles, and free fatty acids, many of which are elevated in PE [11,12]. Activation occurs when these stimuli are recognized by cytosolic sensor proteins, leading to the recruitment of the adaptor protein apoptosis-associated speck-like protein containing a caspase recruitment domain (ASC) and pro-caspase-1. Once activated, caspase-1 promotes the maturation of the pro-inflammatory cytokines IL-1β and IL-18, amplifying the inflammatory response. In this context, UA, traditionally considered a biomarker of PE, has recently been proposed as a potential activator of the NLRP3 inflammasome. Although current evidence remains limited, this suggests a possible molecular mechanism by which UA may contribute to the development of PE. This review aims to examine and critically analyze the existing literature linking UA with both PE and inflammasome activation, to better understand its potential pathogenic role, clarify areas of uncertainty, and highlight future directions for research in this emerging area.

## 2. Methods

A narrative review was conducted to explore the role of UA in pregnancy and PE, with a specific focus on its relationship with the inflammasome. The literature search was carried out using the PubMed database and was limited to studies published between 2005 and 2025.

The search strategy involved the use of the following keywords and MeSH terms: “uric acid”, “pregnancy”, “preeclampsia”, “inflammasome”, “NLRP3”, and “inflammation”, combined using Boolean operators (AND, OR) to refine the results. Additionally, the reference lists of the included articles were reviewed manually to identify further relevant studies that may not have been captured in the initial database query.

Studies were selected based on their relevance to the research question with particular emphasis on those exploring the pathophysiological mechanisms, clinical associations, and potential therapeutic implications of UA and inflammasome activation in pregnancy and PE. The studies included in this narrative review exhibit substantial methodological heterogeneity, ranging from randomized clinical trials to observational studies, meta-analyses, and preclinical animal models. This diversity allows for a comprehensive approach to the topic by incorporating multiple perspectives and levels of analysis. However, it also entails variations in the strength of evidence and the potential risk of bias associated with each study design. While the quality of evidence and risk of bias were not quantitatively assessed using standardized tools, a critical narrative appraisal was conducted. Particular attention was given to the methodological strengths and limitations of each study to interpret the findings within an appropriately contextualized and cautious framework.

## 3. Uric Acid: Metabolism and Physiological Implications

UA is the final product of purine degradation in various organs and tissues, including the liver, intestines, kidneys, muscles, and vascular endothelium [13]. Purines can be endogenous, derived from the breakdown of nucleic acids, or exogenous, originating from protein-rich foods such as fatty meats, organ meats, and seafood, as well as from fructose found in fruits, processed foods with added sugars, and alcohol [14,15]. The purines adenine and guanine are degraded through different pathways to a common intermediate, xanthine, which is subsequently converted into UA by the enzyme xanthine oxidoreductase (XOR). XOR is a metallo-flavoprotein that exists in two forms: xanthine dehydrogenase (XDH) and xanthine oxidase (XO). Both forms catalyze the conversion of xanthine to UA. XDH preferentially uses NAD^+^ and produces NADH, whereas XO uses O_2_ and generates superoxide anion (O_2_^•−^) and hydrogen peroxide (H_2_O_2_) when catabolizing purines. XDH/XO conversion was observed to occur through reversible oxidation in a variety of hypoxic/ischemic and other pathological conditions, but an irreversible proteolytic conversion has also been described in some prolonged ischemic conditions [16].

Blood levels of UA are determined by the balance between its endogenous production, dietary intake, and the rates of renal and intestinal excretion. Intestinal degradation accounts for one-third of UA elimination, while urinary excretion accounts for the remaining two-thirds [17]. In the intestinal tract, UA is transformed into ammonia and carbon dioxide by colonic bacteria. In the kidneys, nearly all UA is filtered by the glomeruli, with post-glomerular reabsorption and secretion regulating the amount eliminated in the urine. In humans, net reabsorption occurs. Approximately 88 to 93% of the filtered UA is reabsorbed by the renal tubules, and only 7 to 12% is excreted in the urine [18]. These mechanisms involve several transporters responsible for UA reabsorption and secretion, predominantly located in the proximal convoluted tubule of the human kidney [19]. Although the process of UA excretion in the intestine is less well characterized, various transporters involved in this process have also been identified in intestinal epithelial cells [20,21].

UA is one of the most abundant antioxidant molecules in humans, with a potent ability to scavenge peroxynitrite, nitric oxide (NO), and hydroxyl radicals, thereby preventing protein nitration and lipid peroxidation [22,23,24]. In this regard, it has been demonstrated in the UOX+/− mouse model, which has only one functional copy of the urate oxidase gene and therefore moderately elevated UA levels, that these mice show an increased lifespan. This longevity is associated with reduced protein nitration and lipid peroxidation in muscle and brain tissues under oxidative stress, suggesting that UA protects cells by scavenging harmful free radicals [22]. Under normal conditions, UA significantly contributes to the total plasma antioxidant capacity, providing defense against oxidative stress. In this regard, it has been described that UA may have protective effects on the cardiovascular system, in various neurodegenerative diseases, in cancer prevention, and during aging [25]. On the other hand, elevated levels of UA can have a prooxidant and pro-inflammatory effect, causing damage to multiple systems throughout the body. Several clinical and epidemiological studies have shown the relationship between UA and various diseases, including cardiovascular disease, metabolic syndrome, and kidney disease, which may outweigh its potential benefits [26].

Elevated blood levels of UA can also be observed in purine-rich and high-fructose diets, alterations in purine metabolism, accelerated purine degradation in states of high cell turnover, and decreased renal excretion [27,28,29,30]. When its concentration exceeds the solubility threshold, it precipitates as crystalline monosodium urate (MSU) in joints, kidneys, and other tissues, triggering inflammation and cellular damage [31]. Both MSU crystals and soluble UA can alter the function of immune cells, such as macrophages, monocytes, neutrophils, and T cells, resulting in a persistent inflammatory state and cellular damage [32]. Furthermore, elevated serum UA levels can impair endothelial function by inducing oxidative stress. It enters endothelial cells via specific transporters, causing mitochondrial dysfunction, superoxide generation, and NO depletion [33,34]. Consistent with these mechanisms, studies in hyperuricemic rats have shown increased oxidative stress and endothelial dysfunction, accompanied by mitochondrial impairment closely linked to reduced ATP production and decreased activity of key Krebs cycle enzymes [33]. Additionally, it can activate the renin–angiotensin system at the vascular level by stimulating angiotensin II production and promoting vascular smooth muscle cell proliferation [35]. Moreover, serum UA can upregulate aldose reductase expression in the endothelium, activating the polyol pathway and promoting endogenous fructose generation, which subsequently undergoes metabolism that increases UA generation, thereby creating a self-amplifying cycle that may exacerbate endothelial dysfunction [36].

These findings highlight the complex role of UA in human health. While it serves as a powerful antioxidant under normal conditions, elevated levels may contribute to oxidative stress, inflammation, and endothelial dysfunction. Its capacity to impair vascular function and immune response underscores its pathological relevance. The regulation of serum UA levels may therefore represent a critical therapeutic target in preventing and managing associated diseases.

## 4. Uric Acid in Pregnancy

During pregnancy, the placenta and the developing fetus significantly contribute to UA production. The placenta, an organ undergoing extensive cellular turnover, constitutes a critical source of purines, which are metabolized into UA via the enzymatic activity of XDH/XO. This mechanism has been previously implicated in the development of hyperuricemia and in the pathological role of XO activity in PE [37].

Beyond its contribution to UA synthesis through purine metabolism, the placenta serves as a significant site of fructose biosynthesis during early pregnancy. Elevated placental fructose leads to increased adenosine monophosphate catabolism through the XDH/XO pathway, raising local UA levels. Notably, fructose concentrations in coelomic and amniotic fluids exceed those in maternal serum between the 5th and 12th weeks of gestation, underscoring its importance in early pregnancy [38]. Fructose is synthesized via the polyol pathway, activated by aldose reductase under low-oxygen conditions typical of the first trimester [39]. Animal studies support this mechanism, showing that maternal high-fructose diets increase placental UA, an effect reversed by allopurinol, implicating placental XO activity in UA overproduction [40].

Despite the substantial increase in placental UA production during early pregnancy, maternal serum UA concentrations in the first and second trimesters are consistently lower than those observed in nonpregnant individuals. Longitudinal studies have provided critical insights into the dynamics of serum UA levels throughout gestation. One such study evaluated serial changes in UA concentrations before conception, at regular intervals during pregnancy, and 12 weeks postpartum [40]. This study demonstrated considerable interindividual variability at each gestational stage while highlighting those individuals who maintained their relative position within the UA distribution throughout pregnancy. Specifically, women with initially elevated UA levels remained in the upper range, whereas those with lower initial levels remained in the lower range [41]. Additionally, a cross-sectional study of 1312 pregnant women, categorized by trimester, compared serum UA levels to those of a matched cohort of nonpregnant women. This analysis revealed significantly lower UA concentrations in pregnant women, ranging from 122 to 297 µmol/L in the first trimester, 129–327 µmol/L in the second trimester, and 147–376 µmol/L at term [42]. A more recent longitudinal study examined UA levels before conception and at four-week intervals throughout gestation, enabling the establishment of more precise reference ranges for this parameter at each stage of pregnancy [43]. Consistent with earlier studies, UA concentrations were found to decrease in the early weeks of pregnancy relative to preconception levels, reaching a nadir between gestational weeks 16 and 20, with a value of 207 µmol/L [193–235]. Following this period, UA levels progressively increased, reaching 281 µmol/L [245–321] between weeks 36 and 40 of gestation [43]. These findings underscore a more pronounced rise in UA levels during the third trimester of pregnancy, along with significant interindividual variability at each gestational stage.

Although the specific mechanisms underlying the decrease in serum UA levels during the first half of pregnancy are not fully understood, this phenomenon is attributed to physiological adaptations characteristic of pregnancy, including hemodilution due to increased plasma volume, enhanced renal clearance, and the uricosuric effects of elevated estrogen levels. Notably, renal blood flow and the glomerular filtration rate (GFR) increase significantly, with documented rises of approximately 50–85% and 40–65%, respectively, by midge station [44]. These adaptations, along with a decrease in tubular reabsorption, may enhance UA clearance, leading to lower serum levels during the first two trimesters of pregnancy. In this context, it has been demonstrated that estrogens can regulate the expression of various UA transporters in the proximal tubule, reducing their reabsorption and increasing their secretion [45]. Consistent with this, studies in ovariectomized mice showed that estradiol and progesterone reduced the protein levels of key renal urate transporters [46]. This suggests that such mechanisms may also contribute to the observed decrease in serum UA levels during the early stages of pregnancy. In contrast, during late pregnancy, as noted in previous studies, a sharp increase in serum UA occurs, possibly indicating that the renal excretion is not sufficient to clear the increasing production of UA from the fetus and the placenta.

## 5. Hyperuricemia and Preeclampsia

The association between PE and elevated serum UA levels was first described in the early 20th century [47]. Since then, extensive research has been conducted to evaluate serum UA concentrations in women with PE compared to normotensive pregnant women [48]. Although serum UA levels are generally higher in preeclamptic women than in those with uncomplicated pregnancies, it is important to note that not all women with the disease exhibit hyperuricemia. The proportion of women with PE who present with hyperuricemia varies across studies, with estimates ranging between 40% and 90% [49,50,51]. This variability is partly due to the lack of a standardized criterion for defining hyperuricemia during pregnancy, as systemic UA levels fluctuate throughout gestation, as previously mentioned. Most studies classify the women under investigation by trimesters, so individual variability is compounded by the changes that occur in this parameter as pregnancy progresses.

Elevated UA levels in PE were initially attributed to impaired kidney function. The disease is associated with endothelial dysfunction and reduced renal perfusion, leading to a decreased GFR [52]. This reduction in the GFR can limit the kidney’s ability to excrete UA, resulting in its accumulation in the bloodstream. Robert et al. [49] conducted a study to evaluate the impact of altered renal function on UA levels in women with PE, both with and without hyperuricemia at delivery. Women with PE and hyperuricemia (HPU) exhibited elevated UA levels from the early weeks of pregnancy, with a more pronounced increase after week 25 compared to women with PE without hyperuricemia (HP). However, after adjusting for creatinine values, the sharp increase observed after week 25 in the HPU group was no longer evident, although UA levels remained consistently higher than in the HP group. This finding suggests that impaired kidney function plays a more significant role in the elevation of UA levels during the second half of pregnancy but does not fully account for the early rise observed.

A significant proportion of UA filtered by the kidneys is reabsorbed by the renal tubules. Disruptions in the regulation of this reabsorption process during pregnancy may contribute to elevated serum UA levels. Dysregulation of UA transporters has been implicated in several hyperuricemia-related conditions, including metabolic syndrome, chronic kidney disease, and cardiovascular disorders [26,53]. However, the role of these transporters in the context of PE remains poorly understood. In this regard, Lüscher et al. [54] demonstrated that one isoform of the GLUT9 transporter, which facilitates UA transport across the basolateral membrane into the bloodstream during reabsorption, can be inhibited by iodide. Their findings suggest that the reduced iodide levels observed in PE may diminish this inhibition, resulting in increased renal UA reabsorption and, consequently, higher circulating UA levels. Additionally, in liver-specific GLUT9 knockout mice, elevated serum UA levels did not affect blood pressure under nonpregnant conditions. However, during pregnancy, these mice developed hypertension starting in the second trimester and exhibited disrupted circadian blood pressure patterns [55]. These results underscore the need for further investigation into the regulation of renal UA transporters in PE. Identifying specific transporters involved in the pathophysiology of PE could offer novel therapeutic targets for managing hyperuricemia and mitigating its clinical impact in affected pregnancies.

In addition to renal factors, increased UA production from placental or fetal tissues, along with heightened XO activity, may also contribute to the hyperuricemia observed in PE. In PE, placental cellular turnover is notably elevated. Under hypoxic conditions, increased cell destruction releases an excess of purines, which serve as substrates for XDH/XO, leading to elevated UA levels [56,57,58]. The fetus is also a potential source of substrates for XDH/XO. Reduced placental blood flow limits nutrient and oxygen delivery to the fetus, leading to fetal hypoxia. In this context, studies on hypoxic fetuses have demonstrated increased blood concentrations of purine metabolites [37]. A recent systematic review by Anessi et al. [59] consistently reported elevated maternal, placental, and fetal XO levels, activity, or expression in women with PE. One proposed explanation for the increase in placental XO is the hypoxic environment that characterizes the placenta in PE, as suggested by Many et al. [60]. Hypoxia is a key stimulus for the conversion of XDH to XO. In addition to producing UA, XO activity generates ROS, such as superoxide and hydrogen peroxide, which contribute to oxidative stress, cellular damage, immune activation, and vascular dysfunction. This oxidative stress plays a central role in the impaired endothelium-dependent vascular relaxation that is characteristic of PE [61].

XO overexpression in PE may also result from inflammatory conditions linked to immune dysregulation and the release of apoptotic trophoblast debris into the maternal circulation. In this regard a study conducted by Santoyo et al. [62] has shown that elevated UA levels in women with high risk of PE were positively correlated with TNF-α and IL-6. In this context, cytokines such as TNF-α, IL-1β, and interferon-γ have been shown to significantly increase XO activity [63]. Additionally, in vitro studies indicate that activated neutrophils can induce the conversion of XDH to XO in endothelial cells, further amplifying oxidative stress [64]. The systemic increase in XO activity observed in women with PE correlates with disease severity and blood pressure levels, highlighting the potential role of XO in the progression of PE. These findings illustrate the complex and multifactorial etiology of hyperuricemia in PE, underscoring the possible role of UA in the disease.

## 6. Diagnostic and Predictive Value of Uric Acid in Preeclampsia

Over the past two decades, numerous studies have analyzed the relevance of maternal UA levels as a biomarker for predicting PE and related maternal and perinatal complications. Several systematic reviews have explored this association from different perspectives.

Cnossen et al. [65] evaluated the predictive accuracy of serum UA levels measured before the 25th week of gestation. They found low sensitivity (below 60%) and variable specificity (77–95%) across studies, concluding that UA in early pregnancy is better at “ruling in than ruling out PE”. In other words, normal or low levels do not exclude the disease due to limited sensitivity.

Thereafter, Koopmans et al. [66] focused on the value of UA in predicting maternal complications, particularly at term (≥37 weeks). Despite heterogeneity among studies regarding population characteristics, test thresholds, testing frequency, gestational age at PE onset and delivery, the interval between testing and outcomes, and reference standards, UA was considered potentially useful in clinical decision making, particularly in guiding obstetric management for women at moderate risk.

Bellos et al. [67] conducted a meta-analysis involving over 39,000 women and confirmed that UA levels were consistently higher in women with PE, especially in early-onset and severe cases, as well as in those with eclampsia or HELLP syndrome. The third-trimester levels demonstrated a diagnostic sensitivity of 76.7% and specificity of 79.6%, while predictive accuracy in the second trimester was more limited. Regarding neonatal outcomes, the biomarker showed variable performance, with AUROC (Area Under the ROC Curve) values between 0.70 and 0.81.

Notably, the predictive performance of UA improves significantly when combined with other metabolic markers. Délic et al. [68] demonstrated that a logistic regression model incorporating UA and urea in the third trimester correctly classified 79.6% of pre-eclamptic cases. When additional parameters, such as platelet count, hematocrit, aspartate aminotransferase (AST), and leukocytes, were added, accuracy improved to 83.8%. Similarly, Zhou et al. [69] found that integrating UA with triglycerides, high-density lipoprotein cholesterol, maternal age, and BMI in the mid-second trimester increased sensitivity for predicting PE to between 80% and 92%, reinforcing the value of UA within a multiparametric panel.

Furthermore, Tang et al. [70] conducted a large retrospective cohort study involving over 20,000 pregnant women and developed multi-stage predictive models using routine antenatal data across all trimesters. Serum UA, measured as early as 5–10 weeks, significantly enhanced model performance, especially when combined with mean arterial pressure, platelet count, and alkaline phosphatase levels in later stages. The predictive accuracy (AUROC) increased from 0.71 at 5–10 weeks to 0.95 at 36–39 weeks. This multi-stage screening approach identified over 94% of future PE cases while minimizing unnecessary interventions in low-risk women, highlighting UA as a valuable, low-cost biomarker in early risk stratification. In line with these findings, a pilot study employing machine learning to predict ICU admission in women with PE identified UA, AST, and BMI as the top three predictive variables. The model achieved strong performance (AUROC 0.91 in cross-validation), further demonstrating that low-cost, routinely available parameters can contribute meaningfully to risk prediction, especially in low-resource settings [71].

A more recent systematic review [72] further confirmed a positive linear relationship between maternal UA levels near delivery and the risk of PE, independent of confounders such as maternal age, gestational age, ethnicity, and socioeconomic status. UA levels were categorized into quintiles based on the control group distribution. Each standard deviation increase in UA was associated with a 21% higher risk of PE. Additionally, a meta-analysis of prospective studies found that women in the highest quartile of UA levels before 20 weeks of gestation had a 1.46-fold increased risk of developing PE, supporting the hypothesis that elevated UA levels may precede the clinical onset of the condition [72]. Complementing these observations, Yue et al. [73] conducted a large retrospective study in a Chinese cohort and found that first-trimester UA levels ≥240 µmol/L were associated with a significantly higher risk of PE (relative risk 1.49), while levels ≥300 µmol/L before 20 weeks were linked to adverse outcomes such as preterm birth and low birth weight. Other studies have also documented this association [51,67]; however, only a few have distinguished between spontaneous and iatrogenic preterm births [51,74,75]. Although the mechanisms underlying spontaneous preterm birth appear to be multifactorial [76], iatrogenic preterm births in PE are well characterized and often result from the need to urgently terminate the pregnancy due to worsening maternal or fetal conditions, such as severe hypertension, fetal growth restriction, or signs of fetal compromise detected through abnormal heart rate patterns. In this context, preeclamptic women with hyperuricemia have been shown to experience higher rates of cesarean delivery, as well as premature births with low birth weight and a small size for gestational age infants. One study reported that the risk of cesarean section in hyperuricemic preeclamptic women was more than doubled compared to those with normal UA levels [51]. Furthermore, among women with PE, 72% delivered via cesarean section, with the rate being significantly higher in the preterm group compared to the term group (78.2% vs. 52.4%) [51]. Kumar et al. [75] also found that most lower-segment cesarean sections were performed in women with high-serum UA. This finding is supported by previous research showing that elevated maternal serum UA levels (≥5.88 mg/dL) were associated with a 2.93-fold increased risk of cesarean section and a higher incidence of low birth weight compared to women with lower UA levels [77]. These findings suggest that hyperuricemia in PE may serve as a marker of disease severity, contributing to increased rates of iatrogenic preterm delivery.

Finally, a recent Mendelian randomization study in European populations offered compelling evidence of a causal association between genetically predicted elevated UA levels and increased PE risk. Among 25 risk factors analyzed, elevated UA showed a statistically significant 21.5% increase in PE risk per standard deviation increase in genetically predicted levels [78].

These findings remained robust across multiple sensitivity analyses, suggesting that UA may not merely reflect disease severity but could contribute directly to PE pathogenesis.

Based on the evidence presented above, although UA may not constitute a definitive standalone predictor, current findings underscore its potential utility in the evaluation of PE risk, as well as its role as a biomarker of disease severity and risk of preterm birth. Its diagnostic performance improves notably when combined with other clinical and biochemical markers, making it a valuable component of multiparametric screening models. Moreover, its low cost, wide availability, and demonstrated association with both maternal and neonatal outcomes make it particularly attractive for early risk stratification, especially in resource-limited settings.

## 7. The NLRP3 Inflammasome as a Potential Contributor to the Pathophysiology of Preeclampsia

### 7.1. NLRP3 Inflammasome and Pregnancy

As mentioned in the Introduction, inflammasomes are key regulators of sterile inflammation. Structurally, inflammasomes consist of three main components: a sensor protein, such as nucleotide-binding oligomerization domain-like receptors (NLRs), absent in melanoma 2 (AIM2)-like receptors, or pyrin, that recognizes specific danger signals; an adaptor protein, apoptosis-associated speck-like protein containing a caspase recruitment domain (ASC), which links the sensor to the effector; and an effector protein, caspase-1, a cysteine protease responsible for processing and activating pro-inflammatory cytokines such as IL-1β and IL-18. The primary function of the inflammasome is to trigger the inflammatory response. The specific sensor protein determines the type of signal that activates the complex. Once activated, the sensor protein interacts with ASC, leading to the recruitment and activation of caspase 1. Activated caspase 1 cleaves pro-IL-1β and pro-IL-18 into their active forms IL-1β and IL-18, which mediate the inflammatory response [79]. Additionally, activated caspase-1 cleaves gasdermin D, forming pores in the cytoplasm and resulting in pyroptosis, a type of lytic cell death mediated by Ninjurin 1 [80].

Several types of inflammasomes have been identified, including AIM2, which detects double-stranded DNA; NLRC4, which responds to bacterial components; and NLRP1 and pyrin, each activated by different pathogens or stress signals. These inflammasomes help the body fight infections, but, if not properly regulated, they can also contribute to chronic inflammatory diseases [79].

Inflammasome components and their downstream effectors, caspase-1 and IL-1β, are expressed by gestational tissues during normal pregnancy and trigger sterile inflammatory cascades during term parturition to eliminate cellular debris and facilitate the reconstruction of the uterine epithelium [80]. The most well-known and widely studied inflammasome is NLRP3. It is characterized by its high expression in innate immune system cells and multiple tissues. NLRP3 can be activated by a broad range of molecules, including both PAMPs and DAMPs.

The sensor molecule NLRP3 and the adaptor protein ASC are expressed in the placenta and chorioamniotic membranes at a moderate level during normal pregnancy and at a higher level during term parturition [81,82,83]. Moreover, NLRP3 is also expressed in the myometrial tissues of women at term [84]. Together with the fact that caspase-1 has also been detected in these locations [85,86,87], this indicates that gestational tissues possess the necessary machinery to initiate inflammasome-dependent immune responses during pregnancy. In this regard, NLRP3 has been implicated in the physiological process of term parturition. Gotsch et al. [88] demonstrated the presence of caspase-1 in the amniotic fluid of women at term, especially those undergoing spontaneous labor. This was accompanied by elevated IL-1β and increased levels of ASC and GSDMD, indicating inflammasome activation. These findings led to the hypothesis that chorioamniotic membranes contribute to labor through the upregulation of inflammasome components, including NLRP3, ASC, and caspase-1, a notion supported by the detection of inflammasome assembly markers like ASC specks and active caspase-1. Further studies extended this involvement to the myometrium and demonstrated that the inhibition of NLRP3 can delay labor in animal models [84,89]. These data suggest that physiological labor at term involves inflammasome activation. While this mechanism is essential for normal parturition, dysregulated or excessive inflammasome activation has been implicated in pathological conditions of pregnancy, including preeclampsia. In this context, aberrant expression or hyperactivation of inflammasome components in gestational tissues may lead to heightened inflammatory responses, endothelial dysfunction, and placental damage, all of which are hallmarks of PE.

### 7.2. Evidence of NLRP3 Activation in Preeclampsia

Increased expression of NLRP3 and related mediators, including caspase-1, IL-1β, and IL-18, has been reported in peripheral blood mononuclear cells [82,90] and placental tissue from PE patients [91,92,93,94,95], compared to normotensive pregnant women. Moreover, specific polymorphisms in the NLRP3 gene have been closely associated with a significantly increased risk of developing PE [96,97].

In line with these findings, Zeng et al. explored NLRP3 inflammasome activation in human samples and in a lipopolysaccharide (LPS)-induced PE model in pregnant rats [98]. They observed marked leukocyte infiltration, particularly macrophages (CD68^+^) and natural killer cells (CD56^+^), in the myometrium and decidua of women with PE, along with the overexpression of NLRP3 and caspase-1. This infiltration was associated with increased levels of pro-inflammatory cytokines IL-1β, TNF-α, and IL-6, and a reduction in the anti-inflammatory cytokine IL-10. In the animal model, LPS exposure reproduced these findings and induced typical PE clinical features such as hypertension and proteinuria.

These results suggest that the uterus, like the placenta, is an active site of inflammation and that the NLRP3 inflammasome plays a central role in amplifying local immune responses, compromising maternal-fetal homeostasis [98]. Providing further evidence of this role, a murine model of PE induced by angiotensin II administration revealed that hallmark PE characteristics, hypertension, proteinuria, and fetal growth restriction, were markedly reduced in NLRP3-deficient mice, emphasizing the critical involvement of the inflammasome in disease progression [99]. In this regard, a recent study demonstrated that metformin, a drug with anti-inflammatory properties, mitigates placental histopathological deterioration and improves pregnancy outcomes in an L-NAME-induced PE rat model by inhibiting NLRP3 inflammasome activation [100]. These findings underscore the key role of placental NLRP3 inflammasome activation in PE, although further research is needed to clarify its mechanisms and clinical implications.

### 7.3. Renal NLRP3 Inflammasome Activation in Hypertension: Implications for Preeclampsia Pathophysiology

Beyond its role in uterine and placental inflammation, the NLRP3 inflammasome has also been implicated in other pathophysiological processes related to PE, such as renal injury, endothelial dysfunction, and hypertension. Its activation, mediated by NF-κB signaling in response to DAMPs, leads to increased production of pro-inflammatory cytokines such as IL-1β and IL-18, which have been observed at elevated levels in both hypertensive patients and animal models [101,102,103]. Moreover, polymorphisms in the *NLRP3* gene have been associated with higher blood pressure and an increased risk of hypertension [29,104]. In animal models, pharmacological inhibition of NLRP3, as well as genetic deletion of its components, has been shown to prevent the development of experimental hypertension, further supporting its role in blood pressure regulation [105].

Activation of the NLRP3 inflammasome has been documented in cardiovascular tissues and in organs central to blood-pressure control, including the kidney. Inflammasome activation in glomerular podocytes and renal tubular epithelial cells is mechanistically linked to glomerular and tubulointerstitial inflammation, leading to proteinuria [106,107,108]. This alteration is highly relevant in the context of PE, because proteinuria, together with hypertension, is one of the most characteristic clinical manifestations of the disease.

Moreover, recent studies provide compelling evidence that the NLRP3 inflammasome plays a pivotal role in sodium reabsorption in the renal tubules and may thereby contribute directly to the pathogenesis of hypertension through the action of its downstream pro-inflammatory cytokines, IL-1 and IL-18.

Zhang et al. [109] demonstrated that IL-1α and IL-1β, derived from the NLRP3 inflammasome, activate IL-1 receptor type 1 (IL-1R1) signaling in the nephron, particularly enhancing sodium reabsorption by upregulating the Na^+^-K^+^-2Cl^−^ cotransporter (NKCC2) in the thick ascending limb of the loop of Henle. This mechanism operates within the context of renin–angiotensin system activation, where IL-1R1 signaling reduces NO bioavailability, a key natriuretic factor that normally inhibits NKCC2 activity. Genetic deletion or pharmacological blockade of IL-1R1 increases renal NO production, reduces NKCC2 function, enhances natriuresis, and protects against hypertension and cardiac hypertrophy. Complementing these findings, Thomas et al. [110] highlighted the role of IL-18, another pro-inflammatory cytokine activated by the NLRP3 inflammasome, in promoting sodium retention during 1K/DOCA/salt-induced hypertension. IL-18 is produced by renal tubular epithelial cells (TECs) and acts in an autocrine/paracrine manner via the IL-18 receptor (IL-18R1), which is strongly expressed on TECs. IL-18R1 is proposed to interact with the Na^+^/Cl^−^ cotransporter (NCC) in the distal convoluted tubule, a key regulator of sodium reabsorption associated with volume expansion in salt-sensitive hypertension. Additionally, studies have shown that MCC950, a specific NLRP3 inflammasome inhibitor, prevents the increase in blood pressure induced by high salt intake [111]. These studies indicate that, by enhancing the activity of key sodium transporters such as NKCC2 and NCC, inflammasome-driven cytokines contribute to sodium retention, extracellular fluid volume expansion, and the development of hypertension. These mechanisms may also be relevant to the pathophysiology of PE, a condition in which sodium retention and edema are prominent clinical features. In this regard, alterations in key sodium transporters, NKCC2, NCC, and ENaC, have been observed in urinary extracellular vesicles from women with PE, indicating increased transporter activity during the disease [112]. Furthermore, in the LPS-induced PE rat model, the renal sodium cotransporters NKCC2 and NCC are upregulated at both the protein and mRNA levels, and exhibit increased phosphorylation and membrane localization, suggesting enhanced functional activity [113].

Taken together, these findings support a model in which inflammasome-driven inflammation and dysregulated sodium transporter activity converge to exacerbate tubular sodium reabsorption, promote extracellular fluid volume expansion, and elevate blood pressure in PE.

### 7.4. NLRP3 Inflammasome as a Mediator of Endothelial Dysfunction: Implications for Preeclampsia Pathophysiology

In animal models of hypertension, such as spontaneously hypertensive rats and Ang II–infused mice, the NLRP3 inflammasome plays a central role in endothelial dysfunction (ED) and vascular remodeling. Activation of NLRP3 in vascular smooth muscle cells promotes phenotypic changes leading to proliferation, fibrosis, and arterial wall stiffening, key drivers of elevated blood pressure [114]. Ang II increases NLRP3 expression, caspase-1 activation, IL-1β production, and reduces p-eNOS-Ser1177, impairing vasodilation. Notably, Nlrp3^−^/^−^ mice are protected from these changes, showing preserved endothelial function and reduced inflammation and oxidative stress. Additionally, NLRP3 increased NADPH oxidase 2/NADPH oxidase 4 and decreased Superoxide Dismutase 2 expression, promoting ROS accumulation and further impairing NO signaling [115]. Therapeutic inhibition of NLRP3 using agents like MCC950 or IL-1β blockade (IL-1Ra) restores eNOS activity and improves vascular function [116]. These findings highlight NLRP3 as a critical link between oxidative stress, inflammation, and vascular damage, positioning it as a promising target for treating hypertension. Similarly, in the context of PE, trophoblastic mitochondrial DNA (mtDNA), particularly under hypoxic conditions (hypo-mtDNA), has been shown to activate NLRP3 and contribute to ED. Hypoxia, a hallmark of the preeclamptic placenta, promotes the release of hypo-mtDNA, which induces inflammation, lowers eNOS expression, and impairs vasodilation. These effects were demonstrated in umbilical cord samples from preeclamptic patients, cultured HUVECs, and pregnant mice. In vivo, injection of hypo-mtDNA into pregnant mice induced vascular inflammation and dysfunction, which were mitigated in NLRP3-deficient animals [117]. Together, these findings support a shared pathogenic mechanism across hypertension and PE, where NLRP3-driven inflammation and endothelial injury represent key therapeutic targets.

## 8. Uric Acid and NLRP3 Inflammasome Activation

Although direct evidence in women with PE remains limited, in vitro studies using trophoblast and monocyte cultures suggest that UA may contribute to the activation of the NLRP3 inflammasome, potentially playing a role in the chronic inflammatory state associated with the disease. Mulla et al. [118] demonstrated that treatment of trophoblasts with MSU crystals led to a reduction in pro-IL-1β protein levels, accompanied by an increase in the expression of active IL-1β, cleaved caspase-1, and ASC. These results indicate that placental cellular stress induced by MSU can activate the NLRP3 inflammasome and initiate a downstream inflammatory cascade.

Similarly, Matias et al. reported that monocytes isolated from women with PE, when stimulated with MSU crystals, exhibited significantly increased expression and secretion of NLRP3, caspase-1, IL-1β, IL-18, and TNF-α compared to monocytes from normotensive pregnant controls [82,119]. Notably, this response was attenuated by silibinin, a compound with recognized antioxidant and anti-inflammatory properties, further supporting the specific involvement of NLRP3 in UA-induced inflammation [119].

Recent evidence has further supported the role of UA in inflammasome activation within placental tissue. A study using placental explants from early-onset PE, late-onset PW, and normotensive pregnancies demonstrated that stimulation with MSU crystals significantly upregulated the expression of NLRP1, NLRP3, HMGB1, caspase-1, IL-1β, and IL-18 at both the mRNA and protein levels. This pro-inflammatory response was most pronounced in early-onset PE explants, which already exhibited elevated basal levels of these markers compared to late-onset and normotensive controls [120]. In this sense, activation of the inflammasome in the placenta could also be associated with adverse perinatal outcomes such as preterm birth, which is characteristic of PE. A recent study by Cavoretto et al. [121] demonstrated that an elevated risk of PE during the first trimester is commonly associated with an earlier onset of spontaneous labor, likely linked to underlying placental dysfunction. Although the exact mechanisms responsible for initiating preterm parturition in humans remain largely unknown, placental inflammation has been suggested as a potential contributing factor [122]. Several studies have shown that amniotic fluid concentrations of IL-1β, IL-18, ASC, and GSDMD are elevated in women with preterm labor and intra-amniotic infection compared to those without this condition, suggesting that the inflammasome may be involved in the pathophysiology of preterm birth in this context [81]. Additionally, a link between NLRP3 inflammasome activation and the mechanisms driving sterile intra-amniotic inflammation-associated preterm birth has also been reported [81]. Altogether, these findings suggest that elevated levels of UA in PE, acting as an endogenous danger signal or alarmin, may activate the placental inflammasome, triggering inflammation, placental dysfunction, and potentially contributing to the onset of preterm birth. Furthermore, early placental dysfunction, as seen in early-onset PE, may lead to emergency obstetric conditions that necessitate preterm or cesarean delivery. These complications are often associated with impaired uterine growth and fetal development, resulting in newborns with low birth weight and classified as small for gestational age [74].

These findings suggest that UA can activate placental inflammasomes, particularly in the early form of disease, contributing to the intensified inflammatory environment characteristic of early-onset PE. Importantly, because elevated UA levels often precede the clinical manifestation of PE, its role in inflammasome activation during early pregnancy may present a window for preventive intervention strategies.

On the other hand, studies in several animal models have demonstrated that UA activates the NLRP3 inflammasome in the kidney, mediating renal damage through the production of cytokines. In this regard, it has been demonstrated that UA-activated NLRP3 inflammasome is associated with inflammation and fibrosis in the kidneys of hyperuricemic rats and mice, leading to glomerular alterations and tubulointerstitial injury [101,102,103]. In human primary renal proximal tubule epithelial cells, UA enhances NLRP3 expression, caspase-1 activation, and the production of IL-1β and ICAM-1, suggesting activation of innate immunity in these cells [104]. Similarly, it has been demonstrated that, in patients with acute kidney injury, renal UA accumulation is associated with inflammasome activation and interstitial inflammation [29]. Consistently, lowering serum urate levels with allopurinol has been shown to reduce renal immune cell infiltration and improve albuminuria, renal fibrosis, and tubular injury [123,124]. Although the study of the relationship between UA and renal inflammasome activation in the context of PE has been limited, the well-established association between this condition and renal damage suggests that such a mechanism could be involved. Given that UA levels are frequently elevated in PE and that UA has been shown to activate the NLRP3 inflammasome in renal cells, it is plausible that UA-mediated inflammasome activation contributes to the renal inflammation and dysfunction observed in this disorder. Further research is needed to clarify the potential role of this pathway in the characteristic renal alterations of PE.

## 9. Uric Acid and NLRP3 Inflammasome as Targets of Preeclampsia

XO inhibitors, such as allopurinol and febuxostat, have been proposed as potential therapeutic agents to reduce cardiovascular and renal events in patients with hyperuricemia by lowering UA levels through inhibition of its production [125,126,127]. Allopurinol has been used during pregnancy in specific contexts, such as fetal cardiac protection and high-risk situations like PE, although its use remains off-label and no major adverse outcomes have been reported in these limited cases [128]. It is classified as Category C by the FDA. Pilot studies in both ovine and rat models have shown that the maternal administration of allopurinol reduces oxidative stress in the fetal cardiovascular system and placenta, improves umbilical blood flow, and mitigates cellular damage following ischemia–reperfusion episodes [129]. In contrast, febuxostat is currently not recommended for use in pregnant women due to insufficient safety data.

Uricosuric agents, such as probenecid and benzbromarone, function by inhibiting renal transporters responsible for urate reabsorption, thereby increasing UA excretion. In addition to their role in managing hyperuricemia, these agents have demonstrated beneficial effects on myocardial function in heart failure and on endothelial function [130,131]. However, their safety and efficacy during pregnancy have not been assessed in human studies.

Finally, other drugs such as statins have been associated with reduced UA levels [132] and, unlike the agents mentioned above, have been investigated for the treatment of PE due to their anti-inflammatory, antioxidant, and endothelial-stabilizing properties. Although some studies suggest potential benefits in women at high risk of developing PE [62,133], the specific role of statins in regulating UA levels in the context of the disease remains unclear. Their use for this indication is not yet fully supported by clinical evidence, underscoring the need for further research.

Currently, there are no specific inflammasome inhibitors approved for the clinical treatment of PE. However, several compounds have demonstrated the ability to inhibit the NLRP3 inflammasome in experimental studies, indicating promising therapeutic potential for this condition (Table 1). Among the most studied inhibitors is MCC950, a selective blocker of the NLRP3 inflammasome. This compound has been shown to significantly reduce blood pressure in animal models of salt-induced hypertension. Its mechanism of action involves preventing the oligomerization of the inflammasome complex, leading to decreased expression of inflammatory cytokines (such as IL-1β and IL-18), as well as reduced renal fibrosis, endothelial dysfunction, and oxidative stress [134]. In the specific context of PE, MCC950 has demonstrated beneficial effects in various experimental models [135,136]. However, its clinical development was halted due to hepatotoxicity in early-phase studies, and it currently lacks regulatory approval for human use [137]. Thus, while MCC950 remains a valuable research tool, its clinical translation is limited by safety concerns, highlighting the need for safer analogs or reformulated versions. Among the indirect inhibitors, Anakinra stands out as a recombinant antagonist of the IL-1α and IL-1β receptor, approved for inflammatory diseases such as rheumatoid arthritis [138]. In animal models of hypertension, treatment with Anakinra has been shown to achieve a significant reduction in blood pressure, as well as a decrease in renal fibrosis and the expression of proinflammatory chemokines [139]. In clinical studies in humans, such as a post hoc analysis of the MRC-ILA Heart trial in patients following acute coronary syndrome, a modest reduction in diastolic blood pressure was observed, but without significant changes in systolic blood pressure and with anti-inflammatory effects confirmed by a sustained reduction in high-sensitivity C-reactive protein levels [140]. These data suggest that Anakinra exhibits a clear antihypertensive effect in animal models and a modest effect in humans, although further clinical trials specifically focused on essential hypertension are needed to confirm its efficacy.

In addition, metformin, a drug widely used for gestational diabetes, has demonstrated anti-inflammatory and NLRP3-inhibitory properties. Studies in PE animal models have shown that metformin not only improves placental morphology and fetal outcomes but also reduces the activation of the NLRP3 inflammasome [100,141], suggesting a dual benefit in PE management through modulation of both metabolic and inflammatory pathways. It is classified as Category B by the FDA, indicating no evidence of fetal risk in humans. Randomized controlled trials, such as the MiG (metformin in gestational diabetes) study, have demonstrated its maternal-fetal safety and efficacy comparable to insulin [142]. These findings open potential avenues for repurposing existing drugs as targeted therapies in PE, particularly in early-onset or high-risk cases.

In summary, the management of PE presents a significant clinical challenge, as it must ensure the safety of both the mother and the fetus. In this context, UA and the NLRP3 inflammasome have emerged as targets in the pathophysiology of the disease. Although therapeutic options specifically targeting these pathways remain limited, compounds such as xanthine oxidase inhibitors, MCC950, and metformin have shown promising results in experimental models. However, their use during pregnancy requires careful evaluation of both safety and efficacy, highlighting the urgent need for well-designed clinical trials. Overall, modulation of UA metabolism and inhibition of inflammasome activation represent innovative and complementary strategies with the potential to improve the management of PE.

**Table 1:** Characteristics of therapeutic agents with potential use in preeclampsia treatment.

AGENT	MECHANISM OF ACTION	LEVEL OF EVIDENCE	USE IN PREGNANCY	REGULATORY STATUS
**ALLOPURINOL**	Inhibits xanthine oxidase, blocking uric acid and reactive oxygen species production [125,126].	Preclinical and some clinical studies in women with preeclampsia (PE) or cardiovascular risk [143,144].	Documented off-label use in pregnant women; no major teratogenic effects reported [144].	Approved for hyperuricemia and gout; off-label use in PE. FDA Category C: Risk cannot be ruled out.
**METFORMIN**	Activates AMPK; improves insulin sensitivity and exerts anti-inflammatory effects. Indirectly inhibits NLRP3 [100,141].	Strong clinical evidence in gestational diabetes; beneficial effects in PE animal models [100,141].	Widely used during pregnancy; considered safe by international guidelines (ACOG, NICE).	Approved by FDA and EMA; widely used in pregnancy. FDA Category B: No evidence of harm in humans.
**MCC950**	Specific NLRP3 inhibitor; blocks inflammasome oligomerization and IL-1β/IL-18 release [134,135,136].	Efficacy shown in animal models of salt-sensitive hypertension and PE. No clinical trials in humans [134,135,136].	Not evaluated in pregnant women; promising preclinical results.	Not approved; in preclinical phase. Clinical development halted due to hepatotoxicity.
**ANAKINRA**	Recombinant IL-1 receptor antagonist; blocks IL-1α and IL-1β signaling, reducing inflammatory cytokine activation and NLRP3 inflammasome downstream effects [139].	Antihypertensive effect in animal models. Modest reduction in diastolic blood pressure and confirmed anti-inflammatory effects in humans following acute coronary syndrome [138,139].	No large studies in hypertensive pregnant women.	Approved for inflammatory diseases such as rheumatoid arthritis. FDA Category B: No evidence of risk in humans, but caution is advised. Off-label use in cardiovascular disease.

## 10. Conclusion and Future Directions

UA has evolved from being regarded merely as a marker in PE to being recognized as a potential active contributor to its pathogenesis. Its utility as a clinical biomarker has been extensively studied, particularly in the second and third trimesters, where elevated levels correlate with increased disease severity, early-onset PE, and adverse perinatal outcomes. However, its diagnostic value as a standalone parameter is limited due to significant interindividual variability during pregnancy, influenced by renal function, placental physiology, and normal gestational adaptations. As a result, its predictive performance improves substantially when incorporated into multiparametric models that combine clinical and biochemical markers—an approach especially valuable in low-resource settings.

The origin of elevated UA levels in PE is multifactorial. It involves reduced glomerular filtration and altered tubular reabsorption, increased placental XO activity under hypoxic conditions, and accelerated cellular turnover in both placental and fetal tissues. These mechanisms not only explain hyperuricemia but also link UA directly to the pro-inflammatory state characteristic of PE.

**Figure 1:**
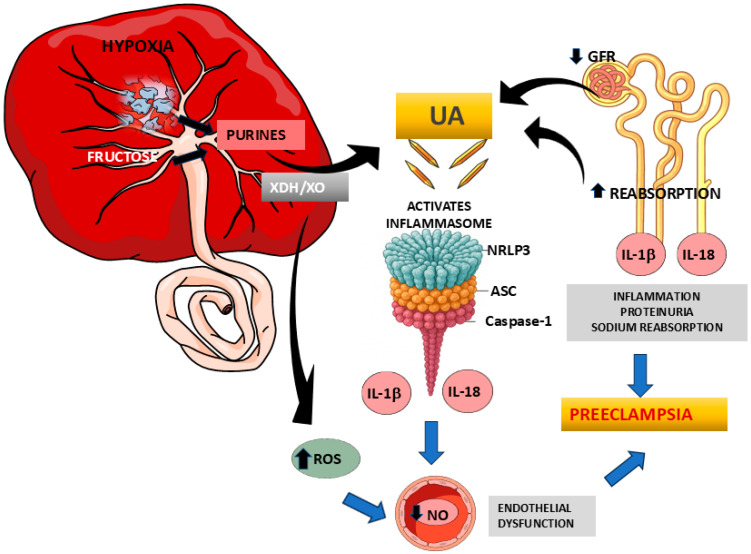
Pathophysiological role of uric acid in preeclampsia. Schematic representation of the relationship between elevated uric acid (UA) levels and the pathophysiology of preeclampsia. During pregnancy, hyperuricemia may result from increased production of placental debris and enhanced fructose metabolism associated with placental hypoxia, which promotes xanthine oxidase (XO) activity and increases reactive oxygen species (ROS). UA levels may also rise due to decreased renal clearance, secondary to reduced glomerular filtration and increased tubular reabsorption. The accumulation of uric acid, along with other damage-associated molecular patterns (DAMPs), activates the NLRP3 inflammasome, leading to elevated levels of inflammatory cytokines such as IL-1β and IL-18. These factors, combined with oxidative stress, contribute to endothelial dysfunction and impaired renal function (including decreased glomerular filtration rate, increased sodium reabsorption, and proteinuria), ultimately promoting the development of the clinical syndrome of preeclampsia.

Importantly, both soluble UA and monosodium urate crystals can activate the NLRP3 inflammasome, a cytosolic multiprotein complex of the innate immune system. Activation of NLRP3 in the placenta, uterus, kidneys, and maternal endothelium leads to the production of pro-inflammatory cytokines (IL-1β and IL-18) and triggers processes such as endothelial dysfunction, sodium retention, and hypertension. This mechanistic link between UA and NLRP3-mediated inflammation highlights the active role of UA in PE pathogenesis, beyond being a simple marker of renal dysfunction (Figure 1).

Therapeutically, this understanding has prompted investigation into agents that can modulate these pathways. XO inhibitors like allopurinol (used with caution in pregnancy) have shown potential in reducing UA levels and oxidative stress. Furthermore, direct NLRP3 inflammasome inhibitors and anti-inflammatory agents, such as MCC950 and metformin, have demonstrated efficacy in animal models by improving placental histopathology, reducing inflammatory cytokine production, and enhancing pregnancy outcomes. However, clinical application in humans and pregnant women requires well-controlled trials to confirm safety and efficacy.

In conclusion, UA is an accessible and clinically relevant biomarker for PE, whose value increases when interpreted within the broader pathophysiological framework involving placental dysfunction, immune activation, and vascular injury. Its role in triggering or amplifying NLRP3 inflammasome activation opens new therapeutic strategies. If validated in clinical studies, targeting UA metabolism and inflammasome signaling could represent a novel and promising strategy in the management of this complex and high-risk obstetric condition.
